# Activity of the antiarrhythmic drug amiodarone against *Leishmania* (*L.*) *infantum*: an in vitro and in vivo approach

**DOI:** 10.1186/s40409-018-0166-7

**Published:** 2018-10-25

**Authors:** Erika G. Pinto, Andre G. Tempone

**Affiliations:** 1 Wellcome Centre for Anti-Infectives Research, School of Life Sciences, University of Dundee, Dundee, UK; 2 Centre for Parasitology and Mycology, Instituto Adolfo Lutz, Avenida Dr. Arnaldo, 351, 8°, Andar. Cerqueira César, São Paulo, SP CEP 01246-902 Brazil

**Keywords:** Leishmania, Visceral leishmaniasis, Amiodarone, In vitro, In vivo

## Abstract

**Background:**

Considering the high toxicity and limited therapies available for treating visceral leishmaniasis (VL), the drug repositioning approach represents a faster way to deliver new therapies to the market.

**Methods:**

In this study, we described for the first time the activity of a potent antiarrhythmic, amiodarone (AMD), against *L.* (*L.*) *infantum* and its in vitro and in vivo activity.

**Results:**

The evaluation against promastigotes has shown that amiodarone presents leishmanicidal effect against the extracellular form, with an IC_50_ value of 10 μM. The activity was even greater against amastigotes in comparison with promastigotes with an IC_50_ value of 0.5 μM. The selectivity index in relation to the intracellular form demonstrated that the antiparasitic activity was approximately 56 times higher than its toxicity to mammalian cells. Investigation of the in vivo AMD activity in the *L. infantum*-infected hamster model showed that 51 days after the initial infection, amiodarone was unable to reduce the parasite burden in the spleen and liver when treated for 10 consecutive days, intraperitoneally, at 50 mg/kg/day, as determined by qPCR. Although not statistically significant, AMD was able to reduce the parasite burden by 20% in the liver when treated for 10 consecutive days, orally, at 100 mg/kg/day; no reduction in the spleen was found by qPCR.

**Conclusions:**

Our findings may help further drug design studies seeking new AMD derivatives that may provide new candidates with an in vitro selectivity close to or even greater than that observed in the prototype delivering effectiveness in the experimental model of VL.

## Background

Leishmaniasis is a neglected tropical disease (NTD) caused by the protozoan parasite *Leishmania spp*., associated with underdeveloped and/or developing countries, presenting infections that manifest especially in tropical or subtropical climates and, consequently, reaching less favored population [1]. Leishmaniasis is called neglected, amongst others factors, because it does not attract the interest of the pharmaceutical sector, which does not consider the market potential sufficient for the investment necessary to develop new drugs [2–4].

Leishmaniases affect a total of 98 countries, 3 territories and 5 continents, presenting endemic transmission and totaling more than 58,000 cases of visceral leishmaniasis and 220,000 cases of cutaneous leishmaniasis per year. There is also an overall global estimate of 0.2–0.4 million cases for the visceral form and 0.7–1.2 for the cutaneous form, making leishmaniases one of the six major endemics for the World Health Organization (WHO) [5, 6].

The chemotherapy for leishmaniases is limited and generally ineffective. The arsenal available is based on a few drugs such as pentavalent antimonial, amphotericin B and miltefosine, which in most cases result in severe side effects and potential treatment abandonment, enabling the appearance of resistant strains [7]. It is also important to highlight that all the drugs cited above were introduced in the leishmaniasis clinic as a result of drug repositioning strategy.

The search for new drugs may require long-term study, large financial resources and a high investment risk. In view of the problematic treatment of visceral leishmaniasis, the screening of drugs developed for another purpose, that is, drugs already available in the clinic to treat other diseases, has been presented as one of the fastest and most effective approaches for introducing new therapies, known as drug repurposing. The repositioning of FDA-approved drugs also stands out as a strategy of lower cost in the medium term with vast examples in the therapy of leishmaniasis [7, 8].

The drug chosen in this study was amiodarone (AMD), which was first synthesized as a coronary vasodilator more than 50 years ago and has been widely used as a potent antiarrhythmic [9]. Additionally, AMD activity has been previously reported within in vitro studies using cutaneous forms of leishmaniasis [10–12].

## Methods

### Drugs and chemicals

3-[4,5-Dimethylthiazol-2-yl]-2,5-diphenyl tetrazolium bromide (Thiazol blue; MTT), M-199 medium, miltefosine, RPMI-PR-1640 medium (without phenol red) and sodium dodecyl sulfate (SDS) were purchased from Sigma–Aldrich (St Louis, MO). Amiodarone was kindly donated by Prof. Dr. Humberto Gomes Ferraz (University of Sao Paulo - Brazil).

### Bioassay procedures

BALB/c mice and Golden hamsters (*Mesocricetus auratus*) were obtained from the animal breeding facility at Adolfo Lutz Institute (São Paulo, Brazil). The animals were maintained in sterilized cages with water and food given ad libitum. Animal procedures were performed with the approval of the Research Ethics Commission (CEUA IAL 04/2011) and in agreement with the Guidelines for the Care and Use of Laboratory Animals from the National Academy of Sciences.

### Parasites and mammalian cells maintenance

*Leishmania* (*L.*) *infantum* (MHOM/BR/1972/LD) promastigotes were maintained in M-199 medium supplemented with 10% fetal bovine serum (FBS) and 0.25% hemin at 24 °C. The *L.* (*L.*) *infantum* amastigotes were maintained in golden hamsters for up to 60–70 days post-infection (d.p.i.) and isolated by differential centrifugation. NCTC (clone 929) murine conjunctive cells were maintained in RPMI-1640 (without phenol red) and supplemented with 10% FBS at 37 °C in a humidified incubator containing 5% CO_2_.

### In vitro cytotoxic concentration (CC_50_) against mammalian cells

The 50% cytotoxic concentration (CC_50_) was determined in NCTC clone 929. NCTC cells were counted in a Neubauer hemocytometer and seeded at 6 × 10^4^ cells per well in 96-well microplates at 37 °C in a 5% CO_2_ incubator. AMD was then added in serial dilutions and tested for 48 h; miltefosine was assayed as the standard drug. The cellular viability was determined by MTT assay at 570 nm [13]. For selectivity index (S.I.) the following equation was employed: S.I. = CC_50_ NCTC cells / IC_50_ amastigotes.

### In vitro inhibitory concentration (IC5_0_) against promastigotes and amastigotes

To determine the 50% inhibitory concentration (IC_50_) against *L.* (*L.*) *infantum*, promastigotes were counted in a Neubauer hemocytometer and seeded at 1 × 10^6^ cells per well in 96-well microplates using miltefosine as the standard drug. AMD was added in serial dilutions and kept for 48 h at 24 °C until parasite viability had been determined by the MTT assay [13]. For IC_50_ determination against amastigotes, peritoneal macrophages were obtained by washing the peritoneal cavity of BALB/c mice with medium and seeded at 1 × 10^5^ cells/well for 24 h. Amastigotes were isolated from previously infected hamsters spleens, separated by differential centrifugation and added to the macrophages at a ratio of 1:10 (macrophage/amastigotes). Non-internalized parasites were removed by washing once with medium; and the cells were then incubated with AMD for 120 h at 37 °C in an incubator under 5% CO_2_, using miltefosine as the standard drug. At the end of the assay, the cells were fixed in methanol, stained with Giemsa and observed under a light microscope to determine the number of intracellular parasites. The number of amastigotes was determined in 400 macrophages from the drug-treated and control wells [14].

### In vivo anti-*Leishmania* activity

The efficacy of AMD treatment (oral and intraperitoneal) was determined using young male golden hamsters previously infected (i.p. route) with *L.* (*L.*) *infantum* amastigotes (1 × 10^8^/animal). Forty days post infection (d.p.i.), the hamsters (*n* = 5/group) were treated intraperitoneally and orally for ten consecutive days with AMD at 50 mg/kg/day and at 100 mg/kg/day, respectively. The control group was treated with vehicle only. The animals were euthanized using CO_2_ 50 d.p.i. and the parasite burden was evaluated by real time PCR using RNA samples obtained from the spleen and liver fragments, according to a standardized method published by Reimão and co-workers [15]. The susceptibility of *Leishmania infantum* to pentavalent antimony (Glucantime®) was previously determined in a hamster model [16].

### Statistical analysis

All data obtained have been reported as the mean of two/three independent assays. The IC_50_ values were calculated using sigmoid dose-response curves by the software Graph Pad Prism 5.0 (GraphPad Software Inc., La Jolla, CA), and the 95% confidence intervals were included in parentheses. ANOVA was used for statistical analysis.

## Results

### In vitro anti-*Leishmania* activity (IC_50_) and cytotoxicity concentration (CC_50_)

The AMD assay performed against *L.* (*L.*) *infantum* promastigotes showed, after 48 h of incubation, an IC_50_ value of 10.5 μM. The drug was able to reduce by 100% the promastigotes viability at the highest concentrations. In order to determine the respective selectivity index for further intracellular investigation (amastigotes), the cytotoxicity assay was performed using NCTC cells, which presented a CC_50_ value of 30.9 μM versus an IC_50_ value of 0.55 μM for the amastigote form. AMD also exhibited a selectivity index of 56, relative to the intracellular form of the parasites. Miltefosine was used as the standard drug and the values are shown in Table 1.

**Table 1:** In vitro IC_50_ against promastigotes and amastigotes of *Leishmania*, CC_50_ against NCTC cells and SI

Drugs	Promastigotes IC_50_ (μM) (95%CI)	Amastigotes IC_50_ (μM) (95%CI)	Cytotoxicity CC_50_ (μM) (95%CI)	SI
Amiodarone	10.5 (9.5–11.7)	0.5 (0.1–2.0)	30.9 (12.5–76.1)	56.2
Miltefosine	16.8 (15.4–17.5)	17.8 (11.6–24.6)	122.0 (94.8–157.0)	6.8

*IC*_*50*_ 50% inhibitory concentration, *CC*_*50*_ 50% cytotoxicity concentration, *95% C.I*. 95% confidence interval, *S.I* selectivity index

### In vivo anti-*Leishmania* activity

Considering the in vitro activity of AMD against intracellular amastigotes, the next step was to perform an in vivo experiment using the *L. infantum*-infected hamster model, in which after 40 d.p.i. the animals were treated for 10 consecutive days by two different routes of administration: intraperitoneal at 50 mg/kg/day and oral at 100 mg/kg/day. After 51 days of the initial infection, the animals were euthanized, spleen and liver fragments removed and the parasite burden was determined by qPCR.

**Fig. 1:**
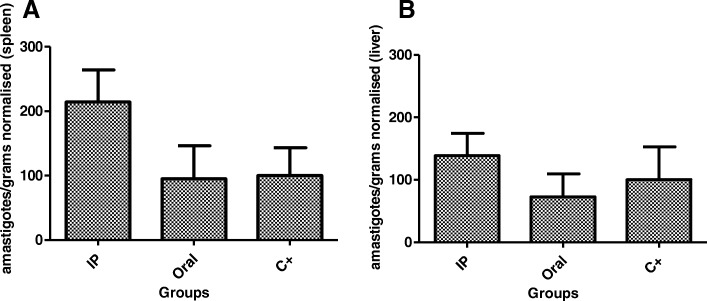
In vivo evaluation of AMD in *Leishmania*-infected hamsters. The drug was administered for ten consecutive days at 50 mg/kg (i.p.) and 100 mg/kg (p.o.). Real-time PCR quantification (RNA amastigotes) of parasite burden in spleen (**a**) and liver (**b**). Data was normalized based in the vehicle group

The results demonstrated that AMD was not able to reduce the parasite burden in either the spleen or liver after intraperitoneal treatment. Although not significant (*p* > 0.05) a 114% increase in spleen infection and 38% in liver infection was observed in relation to the control group. In view of the oral treatment, AMD was also unable to reduce the infection in the spleen (5% reduction); however, AMD was able to reduce the parasite burden by 20% in the liver, although without statistical significance (*p* > 0.05) (Fig. 1). The data below refer to a representative assay of two distinct experiments.

## Discussion

Given the drug repositioning strategy and its great impact on leishmaniasis therapies – as demonstrated by the introduction of antimony, pentamidine, amphotericin B, azoles (ketoconazole, itraconazole, etc.), used initially to treat fungal infections [17], as well as miltefosine, developed for cancer treatment [18] – the drug amiodarone was chosen for the experimental trials due to its promising and unprecedented in vitro activity against *L.* (*L.*) *infantum*.

Considering the in vitro models, AMD showed a high selectivity index, demonstrating that the antiparasitic activity was approximately 56 times higher than the toxicity against mammalian cells. Additionally, AMD was about eight times more selective when compared to miltefosine, the standard oral treatment for VL in India. Based on the IC_50_ values against intracellular amastigotes, AMD was found to be approximately 32 times more potent than miltefosine. These findings corroborate the data described by Serrano-Martín et al. [10], in which they not only demonstrated promising selectivity of the drug AMD against *L. mexicana*, but also observed higher IC_50_ values in amastigotes (8 nM) when compared to promastigotes (900 nM).

The anti-*Leishmania amazonensis* [11], anti-*Leishmania mexicana* [10] and anti-*Leishmania braziliensis* [12] activities presented by AMD were previously described, both under in vitro (promastigotes and amastigotes) and in vivo models. Considering that different species may result in different sensitivities against the same drug [19], it was observed that AMD resulted in very similar IC_50_ values when compared to the species that cause tegumental or visceral diseases. This is exemplified by the finding by Nishikawa et al. [20] that AMD’s anti-*L. amazonensis* activity resulted in an IC_50_ value of 0.46 μM, a fact that is extremely important when developing a drug not just for one disease but for a complex of diseases such as the complex of leishmaniases. However, the data presented in this paper to the best of our knowledge are unprecedented, since no study has been found in the literature demonstrating the potential of AMD against the fatal visceral form of leishmaniasis.

The anti-*Trypanosoma cruzi* activity of AMD has also been demonstrated through both in vitro and in vivo models, showing a mode of action related to mitochondrial damage and inhibition of ergosterol synthesis [21]. Amiodarone has also demonstrated an action that inhibits the calcium channels [22]; data from the literature have shown the activity of calcium channel blockers against amastigote forms of *L.* (*L.*) *infantum* [23]. Data reported by Paniz-Mondolfi et al. [12] demonstrated that AMD induced a “parasitological cure” in a single clinical case of *L.* (*V.*) *braziliensis* when administered at the dose of 1,600 mg/day for the first 4 days, followed by a dose of 800 mg/day for 3 consecutive weeks. Additionally, an important fact to highlight associated with diseases caused by trypanosomatids and amiodarone use includes the drug prescription for chagasic patients that present cardiac compromise.

Taking into consideration the potential of this drug in the literature as well as its in vitro effectiveness, the study was carried out in an experimental hamster model of infection with *L.* (*L.*) *infantum*. Given the WHO criteria for new oral drug candidates for LV [5], the present study approached both oral and intraperitoneal administration. The results clearly demonstrated that after 10 days of treatment AMD was unable to induce any treatment in the target organs (spleen and liver), even when administered by two different routes (oral and intraperitoneal). Among the factors that could have contributed to the lack of effectiveness by the oral route, it is suggested that the absorption of the drug by the gastrointestinal tract is low and variable. Data from the literature report that only 20 to 55% of the drug is found in the circulation after oral administration [24]. In this case, future pharmaceutical formulations could be developed in an attempt to promote a better absorption of the drug, such as the production of AMD as nanocrystal, which favors an increase in the effectiveness of drugs, due to a better bioavailability [25]. Furthermore, the maximum oral dose used, namely 100 mg/kg/day, may not have been sufficient to achieve adequate plasma levels for parasite elimination. The literature describes the Lethal Dose 50% (LD_50_) after intravenous administration as 227 mg/kg in mice [26]; the use of higher doses might have caused toxicity in the animals, making the study inviable.

Another important factor that may have contributed to the lack of effectiveness in the animal model may be related to drug metabolism. AMD has been described as being extensively metabolized by the liver via CYP450, specifically by CYP2C8, resulting in a major metabolite, desethylamiodarone [27]. Although this metabolite continues to exhibit antiarrhythmic activity [28], nothing has been described in the literature as to its anti-*Leishmania* potential, a fact that should be investigated in an attempt to explain the absence of activity against the parasite in the experimental model.

AMD is an FDA-approved drug, is in conformity with Lipinsky’s “Rule of Five” [29] and despite being in clinical use, presents problems related to its long half-life (~ 58 days) [24] and consequent slow excretion may cause toxicity to the organism [30, 31]. Although its pharmacokinetics profile is not ideal, the literature reports the daily use of AMD in patients, justifying in this work the dose regime choice in the experimental model. Considering the regimen adopted for both oral and intraperitoneal routes, it is possible to suggest that the drug may have contributed to animal toxicity, since a two-fold increase of parasite burden in spleen by intraperitoneal route was observed in relation to the control group. To corroborate this result, numerous studies in the literature describe AMD hepatotoxicity [32–34]. It is also important to highlight a case study that reported hepatic failure following AMD intraperitoneal administration at a dose of 750 mg [32]. Additionally, the drug possesses extremely lipophilic properties with consequent accumulation in the liver, which results in tissue levels 500-fold higher in the liver than those found in the circulation [35]. Plomp and co-workers [36] demonstrated the high accumulation of the drug and metabolite in adipose tissues. Moreover, the toxicity expressed at higher doses following intraperitoneal administration of AMD may be associated with the action mode of the drug, which induces mitochondrial stress further aggravated by its metabolite desethylamiodarone [37].

In contrast, the literature provides reports of in vivo efficacy of AMD in a tegumental leishmaniasis model. Serrano-Martín et al. [38] demonstrated that after oral treatment with amiodarone at 50 mg/kg/day in an experimental model of *L. mexicana*, the drug produced superior efficacy compared to Glucantime^®^. However, the authors reported that after drug decrease, reactivation of the disease was observed, indicating therapeutic failure [38]. Given the long half-life of AMD, combination therapy studies have been performed in VL therapy.

Serrano-Martín et al. [38] showed in vivo synergism of AMD when given in combination with miltefosine. The administration of amiodarone at 50 mg/kg/day + miltefosine at 20 mg/kg/day resulted in a “parasitological cure” in 90% of the animals, as evaluated by optical microscopy, PCR and cell culture [38]. Furthermore Anversa et al. [39] showed that amiodarone used either on its own or in combination was unable to stop the development of cutaneous lesions caused by *L. amazonensis*; however, an improvement of pentavalent antimonial activity in the lesions has been observed with no side effects [39].

Additionally, data in the literature indicate that dronedarone may be a potential analog for future in vivo studies against *L.* (*L.*) *infantum* because the compound is: i) a structural analogue of amiodarone; ii) has a lower half-life (~ 18 h); iii) demonstrated in vitro values against *L. mexicana* more promising than amiodarone (promastigotes: 115 nM versus 900 nM/amastigotes: 0.65 nM versus 8 nM) [40].

Finally, studies of drug delivery systems, such as phosphatidylserine-containing liposomes [41], may constitute a very promising alternative to target AMD at lower doses toward organs affected by the parasite, such as the liver, spleen and bone marrow.

## Conclusions

Our study indicates that AMD is an in vitro potent FDA-approved drug against intracellular amastigotes of *Leishmania (L.) infantum* and may represent a lead compound for future synthesis of new analogues. Although the in vitro potency was clearly present in this compound, future analogues should also consider the reduction of the plasma half-life (T_1/2_), since long T_1/2_ values can induce resistant parasites. However, these findings may serve as a basis for drug design studies directed at new AMD derivatives that could provide new candidates with an in vitro selectivity close to or even greater than that observed in the prototype seeking to deliver effectiveness in the experimental model of VL.
